# Influence of an in-plane uniform electric field on 2D exciton states in CdSe nanoplatelets

**DOI:** 10.1039/d5na00378d

**Published:** 2025-07-02

**Authors:** Davit A. Baghdasaryan, Volodya A. Harutyunyan, Hayk A. Sarkisyan, Lyudvig S. Petrosyan, Tigran V. Shahbazyan

**Affiliations:** a Institute of Applied Problems of Physics 25 Hr. Nersisyan St. Yerevan 0014 Republic of Armenia hayk.sarkisyan@rau.am; b Department of Physics, Jackson State University Jackson MS 39217 USA

## Abstract

The influence of an external uniform in-plane electrostatic field on the exciton states in a CdSe nanoplatelet (NPL) is considered theoretically. By considering the jump in permittivity at the NPL-medium boundary, the energy spectrum and spatial distribution of the probability density for free carriers and 2D excitons in the presence of an in-plane electric field are obtained. The Stark shifts for a 2D exciton are calculated, and it is shown that for fields above a certain critical value, the exciton decays into an electron and hole pair. It is shown that the field critical value increases with a decrease in the number of monolayers in the direction of strong NPL quantization. The exciton decay rate dependence on the in-plane electric field has been calculated. The main decay mechanisms have been identified for regions of weak and strong electric fields. For the field values less than the critical exciton radiative decay time, calculations of ionization time *via* tunneling of an exciton are presented. Along with the dependence on the external field, their dependence on the number of monolayers in the direction of strong quantization and the depth of the quantum well NPL in the lateral direction is also shown. For a strong electric field, single-particle states are studied in the NPL plane, and an estimate is given for the tunneling time of electrons through the barrier created by the field for charge carriers in the lateral direction after exciton decay.

## Abstract 2

Theoretical total exciton decay rate in CdSe NPLs in the presence of an external field.

## Graphical abstract

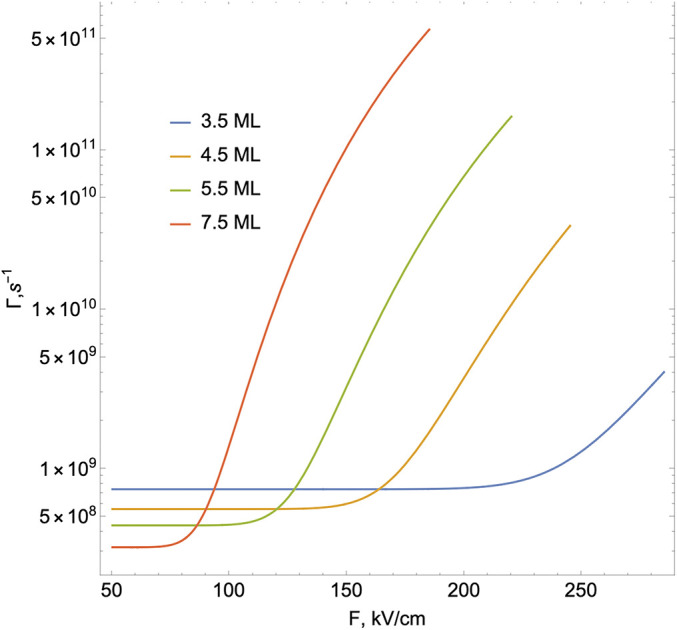

## 1. Introduction

Semiconductor nanoplatelets (NPLs) are quasi-two-dimensional quantum nanostructures with anomalously strong size quantization in one direction, which have attracted intense interest during the past two decades.^1–12^ NPLs exhibit unique optical properties such as an extremely narrow width of the absorption and emission lines, low generation threshold, short photoluminescence time, large values of the exciton binding energy and absorption cross-section, gigantic oscillator strengths, high degree of optical gain, *etc.*^1–18^ which make them very promising structures for a wide range of applications including optoelectronics^5–7,10–24^ and biomedicine.^25–28^ The optical spectra of NPLs, which are based mainly on II–VI compounds, are determined by the excitonic states^20,29–36^ that are characterized by large binding energies *E*^2D^_b,ex_ ∼ 200–300 meV at room temperature,^32,34,35^ as compared to those in bulk CdSe (*E*^3D^_b,ex_ ∼ 15 meV) and quantized film-quantum wells (*E*^QF^_b,ex_ ∼ 60 meV).^32^ The exciton binding energies exhibit strong dependence on the NPL thickness and increase substantially on reducing the number of monolayers (ML).^32,34,35^ Another distinct feature of two-dimensional (2D) excitons in NPLs is the quantization of their in-plane center-of-mass motion due to the in-plane confinement (∼10 nm) of NPLs, which strongly affects their absorption and photoluminescence (PL) spectra.^37^ At the same time, excitonic states that determine the optical spectra in semiconductor structures can be tuned in a wide range by applying a static electric field.^38–46^ In CdSe spherical quantum dots, the quantum-confined Stark effect has been discussed in the studies.^47,48^ Recently, there has been much interest in the effects of the external electric field on the excitonic states in semiconductor NPLs and other mono- and few-layer quasi-2D semiconductor structures.^49–58^ In CdSe NPLs subjected to a uniform electric field, a strong broadening of the absorption band was observed due to quantum-size Stark and Franz–Keldysh effects, which is an order of magnitude larger than that in quantum dots (QDs) made of the same material.^49^ The effect of an electric field on excitonic emission was studied in colloidal CdSe NPLs with a thickness of 5 monolayers and about 170 meV exciton binding energy, where up to a 28% change in radiation rate was observed.^50,51^ In ref. 52, the effect of an in-plane field on exciton binding energies in 2D monolayer and few-layer systems was studied theoretically by analyzing the Schrödinger equation with a field-dependent effective mass using the Rytova–Keldysh potential. In ref. 53–56, it was reported that excitons in these materials have sufficiently large binding energies and, therefore, the electron–hole dissociation is not observed up to electric field strengths of the order of 100 kV cm^−1^. The effect of an axial electric field on excitonic states and the corresponding changes in the linear and nonlinear optical response was addressed in ref. 57 along with the correlating effect of the axial electric field on 2D excitonic states in the NPL plane. The redshift of PL peaks observed experimentally in PbS nanoplatelets–nanosheets in an in-plane field^58^ was attributed to the Stark effect. This work reveals electric field-dependent tuning of charge transfer excitons by up to 100 meV in MoSe_2_/WSe and CdSe/CdS nanoplate heterostructures, offering a pathway for electrically tunable mixed-dimensional excitonic devices.^59^ This study demonstrates Autler–Townes splitting in solution-processed CdSe nanoplatelets, with electric field-driven spectral modulation enabled by femtosecond near-infrared pulses and strongly angle-dependent intersubband transitions.^24^ Most of the aforementioned studies focused on the effect of an external electric field on excitonic emission in 2D materials and NPLs. For many applications, however, a crucial issue is exciton stability, as well as its lifetime dependence on the external field. Recently, field-induced exciton dissociation was observed in monolayer WSe_2_, and the transition from exciton PL to free electron–hole emission was monitored through a sharp change in electric field dependence of the PL time.^56^ In the current paper, we theoretically study the stability of exciton states in CdSe NPLs in an external in-plane electric field by analyzing the PL time's field dependence. Within our approach, the change in the number of monolayers of CdSe NPLs is considered within the adiabatic approximation, while polarization effects in the axial direction are included using the image method. By numerically solving the Schrödinger equation, the dependence of the recombination time on the external field value is calculated, and electric field ranges are determined for each decay mechanism. The corresponding mechanisms of exciton decay are identified in the ranges of external field values. We have shown that for comparatively weak fields, two mechanisms of exciton decay operate: radiative recombination, which depends weakly on the field, and ionization, which is determined by the external field. It is also shown that above a certain critical field value, the optical response is affected by the electron tunneling from the NPL region. The paper is organized as follows. In Section 2, the effect of an in-plane uniform electric field on 2D exciton states in CdSe NPLs is analyzed. In Section 3, the exciton lifetime mechanisms are investigated for NPLs in the in-plane electric field. In Section 4, the free carriers in NPLs in the presence of a strong electric field are studied. Section V concludes the paper.

## 2. Exciton states in NPLs in the presence of an in-plane electrostatic uniform field

In this section, we present our model for 2D excitonic states in NPLs in an in-plane electric field. We assume that the exciton density is sufficiently low such that exciton–exciton interactions are rare. As a result, nonradiative recombination mechanisms arising from these interactions can be safely neglected.^34,56^ Additionally, other nonradiative processes typically become significant at higher exciton densities. The Hamiltonian of an electron and a hole interacting with each other in the presence of an in-plane field has the following form:1
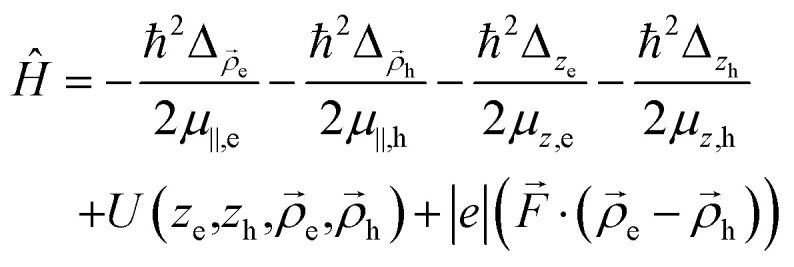
Here 
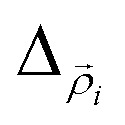
 and *Δ*_*z*_*i*__ are the Laplacian operators for an electron and hole in the *XY* plane and along the stacking direction, respectively, *μ*_‖_*i*__ and *μ*_*z*_*i*__ are the corresponding effective masses for particle *i* = e, h (electron or hole) respectively, 
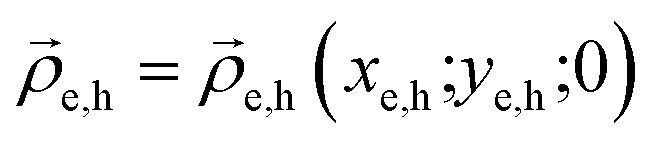
 are the in-plane 2D radius-vectors of an electron and hole, respectively, *e* is the elementary charge, and 
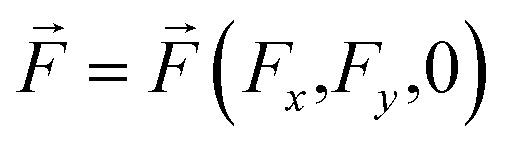
 is the in-plane electric field strength. The potential energy term of an electron–hole pair can be written as:

2 *U*(*z*_e,_*z*_h,*ρ*_) = *U*^e^(*z*_e_) + *U*^h^(*z*_h_) + *U*^e^_conf_(*x*_e,_*y*_e_) + *U*^h^_conf_(*x*_h,_*y*_h_) + *V*(*z*_e,_*z*_h,_*ρ*)

where 
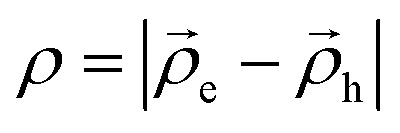
, *V*(*z*_e_,*z*_h_,*ρ*) is the interaction between an electron and a hole, *U*^*i*^(*z*_*i*_) are one-particle confining potential energies appearing due to the conduction and valence band offsets, together with the self-energy correction appearing because of the mismatch of the dielectric constants in the regions of a barrier and a well:^60^

3 *U*^*i*^(*z*_*i*_) = *U*^*i*^_conf_(*z*_*i*_) + *U*^*i*^_self_(*z*_*i*_)

The confining potential defined by the band offset between the NPL and its surrounding material is taken as a finite- and infinite-depth potential well along the *z*-axis and *x*–*y* plane, respectively:^35^4
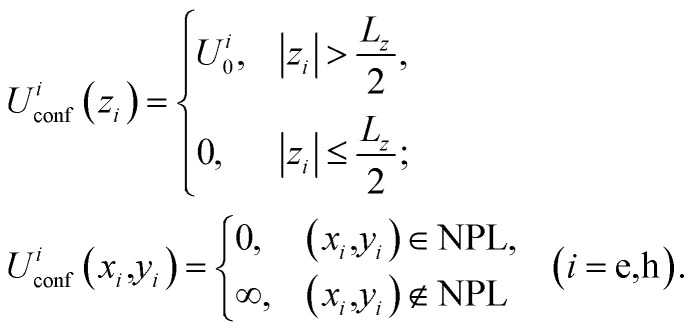
Here *U*^*i*^_0_ ≡ *U*^e,h^_0_ is the depth of the potential well for the electron and hole, respectively, and *U*_self_(*z*) is the self-induced potential for every type of carrier (see, for example, ref. 35, 60 and 61):5
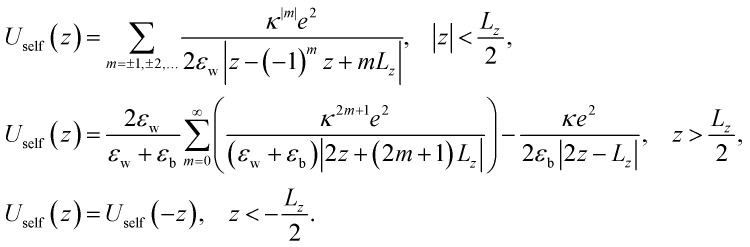
where 
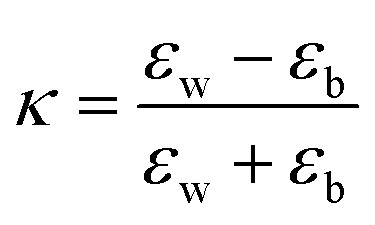
, *ε*_w_ and *ε*_b_ are the dielectric constants of the NPL material and of the spacer, respectively, and *L*_*z*_ is the thickness of the NPL along the quantization direction.

The solution of the Schrödinger equation with Hamiltonian (1) in this case can be obtained with sufficient accuracy within the framework of the adiabatic approximation. Indeed, the energy of the “fast” movement of charge carriers along the quantization *z*-axis will always be significantly greater than the energy of any of the carrier states in the NPL plane. Accordingly, let us write the total wave function of an interacting electron–hole pair in an external field with the following form:6
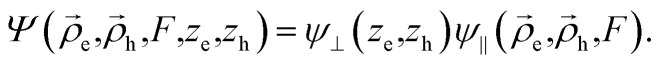


Using the confining potential along the quantization axis from (4) and (5) and separating the *z* variables in (1), we get the wave functions *ψ*_⊥_(*z*_e_,*z*_h_), which in turn will be presented as a product of functions of single-particle states of electrons and holes:^35^

7 *ψ*_⊥_(*z*_e_,*z*_h_) = *ψ*_⊥_(*z*_e_)*ψ*_⊥_(*z*_h_).

After this, by analogy with ref. 35, 60 and 61 the electron–hole interaction potential 
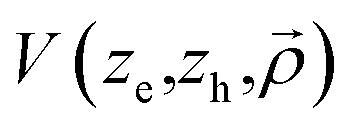
 is represented by averaging over single-particle states (7).8
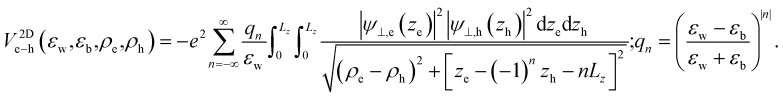


To determine in-plane excitonic states in NPLs, we now obtain the following Hamiltonian:9
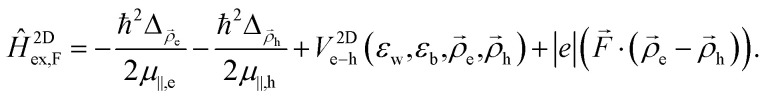


For typical NPLs, the following conditions between the longitudinal *L*_*z*_ and lateral *L*_*x*_, *L*_*y*_ dimensions are always met:^19,20^

10 *L*_*z*_^2^ ≪ *a*_ex_^2^, *L*_*z*_^2^, *a*_ex_^2^ ≪ *L*_*x*_^2^, *L*_*y*_^2^.

Here, *a*_ex_ is the Bohr radius of the exciton. Condition (10) allows us to separate the relative motion of the electron and hole and the motion of the center of mass of the 2D exciton in the *XY* plane.^34,35,62^ Correspondingly, for the definition of full wave functions *ψ*_‖_(*x*_e_,*y*_h_,*F*) = *ψ*_*N*x,*N*_*y*__(*X*,*Y*)*ψ*_ex_(*x*,*y*,*F*) from (6) and energy 
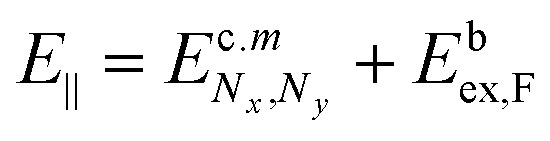
 of an in-plane interacting electron–hole pair we can write the following two equations for center mass and relative motion of an e–h pair, respectively:11
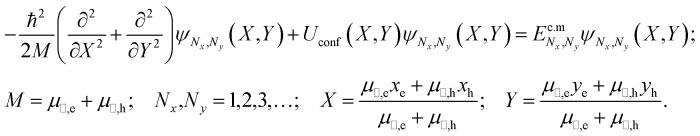
12
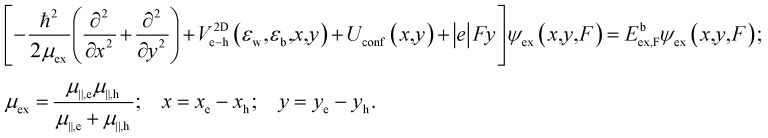
Here, (*μ*_‖_)_e,h_ – effective electron and hole masses in the NPL plane, *V*^2D^_e–h_(*ε*_w_,*ε*_b_,*x*,*y*) is the interaction potential between an electron and hole averaged over single-particle states (see (8)), 
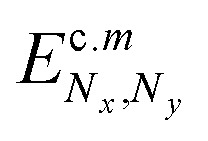
 – energy of motion of the center of mass of a 2D exciton in the *XY* plane, and *E*^b^_ex,*F*_ – energy of the bound excitonic state of an electron and a hole in the NPL plane in the presence of an external field:13
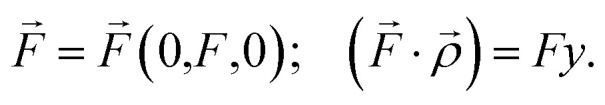


We chose the external field to be directed along the *y*-axis for definiteness. The above reasoning and the corresponding expressions obtained on their basis are general for NPLs that satisfy the conditions presented in the problem. Let us now turn to the consideration of the action of an in-plane uniform electric field on specific structures of CdSe NPLs. In the model, we assume that the band structure and, in particular, the effective mass of the exciton does not change under the influence of an electric field.

**Fig. 1:**
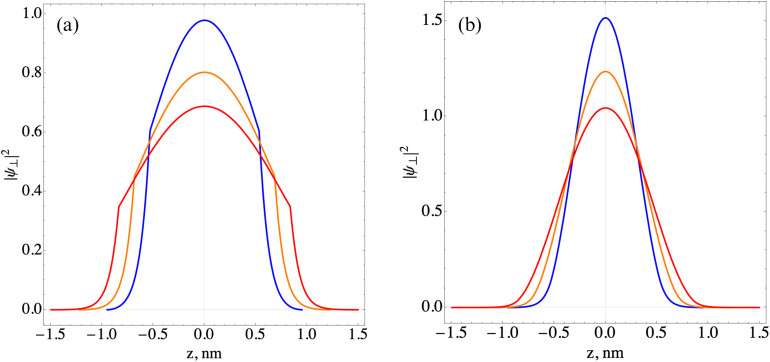
The electron (a) and hole (b) ground state density probability distribution in the axial direction for different NPL thicknesses (*n* = 3.5 (blue), *n* = 4.5 (orange), and *n* = 5.5 (red) ML).

When the external field is oriented according to (13), the field does not have any effect on the states of eqn (7) as they move along the quantization axis (*z*). Accordingly, using the methodology of ref. 35 and 61 we obtain a numerical solution for the wave functions and energies of the lowest states of size quantization of charge carriers along the *z*-axis, which are of real physical interest. Table 1 presents the values of the first two energy levels of size quantization of the electrons 
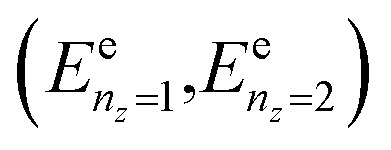
 and the holes 
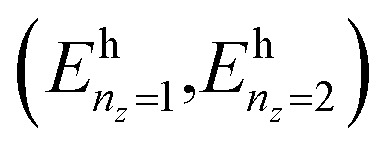
 for different numbers of monolayers (ML) along the quantization axis, and the corresponding wave functions of ground-state charge carriers are shown in Fig. 1.

**Table 1:** Values of the first two levels of size quantization of electrons 
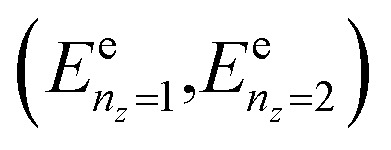
 and holes 
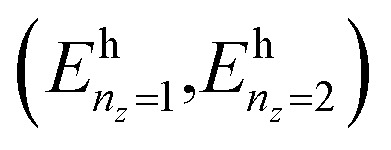
 in a CdSe nanoplate for different numbers of monolayers along the quantization axis (*z*). Data taken from ref. 35

ML	*μ*_*z*,e_/*m*_0_	*μ*_*z*,h_/*m*_0_	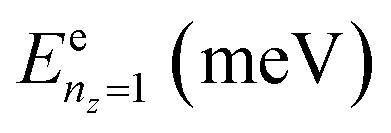	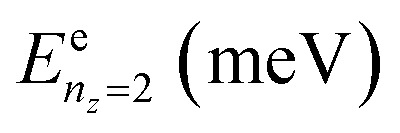	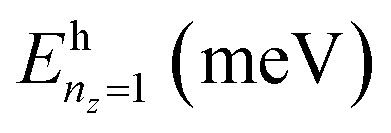	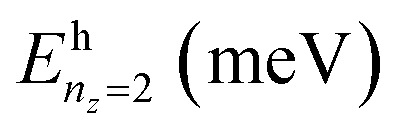
3.5	0.157	0.96	439	—	280	937
4.5	0.144	0.92	341	1700	197	665
5.5	0.138	0.90	277	1330	147	483
7.5	0.130	0.88	197	887	92	294

Here, *m*_0_ is the mass of a free electron. The depths of the confinement potential in the axial direction for an electron and hole are taken to be *U*^e^_0_ = 2 eV and *U*^h^_0_ = 2.5 eV, respectively.^63^

Let us now turn to the consideration of electron–hole states in the plane in the presence of an external field (13). Within our model, the external field does not affect the movement of the 2D exciton center of mass. Solving eqn (11) analytically, for the energy and wave functions of motion of the center of mass of an electron–hole pair in the *XY* plane leads to the following results:14
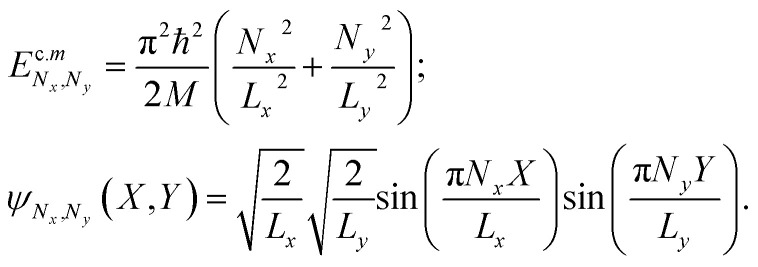


**Fig. 2:**
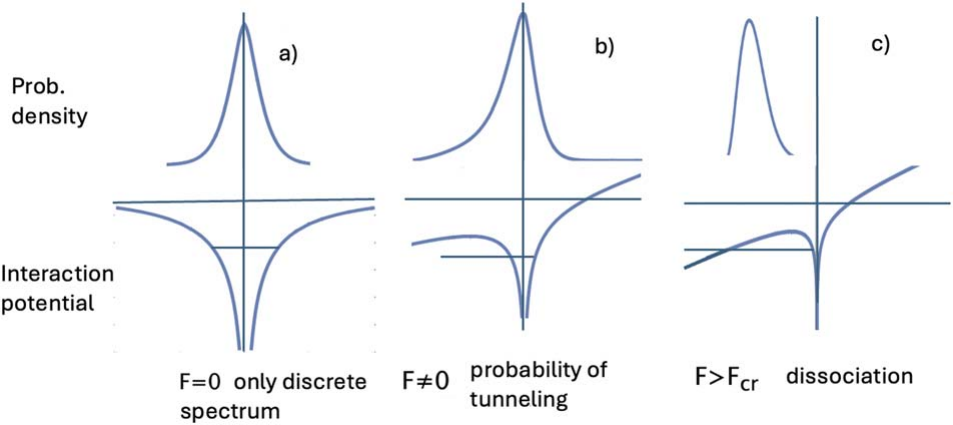
Schematic illustration of 2D exciton states in the absence (a) and presence (b and c) of a static electric field. In the case of the absence of an electric field, only a discrete spectrum is possible with negative energies. In the case of a non-zero electric field (b) dissociation *via* tunneling takes place (b), for the electric field values larger than some critical value, when the dissociation has already occurred.

Eqn (12) can be solved numerically. In the case of *F* = 0, the energy values of the ground exciton states and Bohr radii coincide with the results of the studies.^34,35^ It is clear that the external field affects the shape and behavior of the total potential (Fig. 2). Accordingly, as the numerical solution of eqn (12) shows, this necessarily leads to a change in the position of the energy levels (Stark shift) and a change in the behavior of the wave function of the pair.

**Fig. 3:**
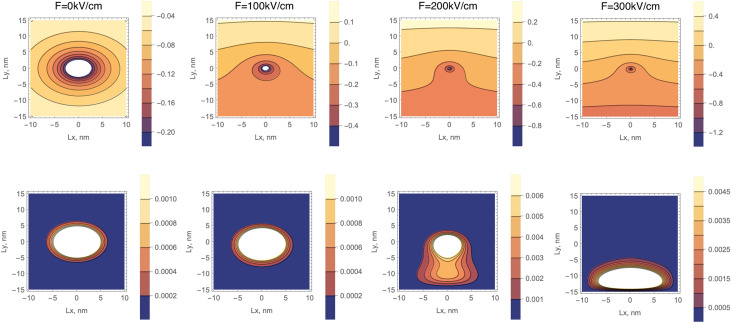
The deformation of the electron–hole interaction potential under the influence of an external electric field (upper row) and redistribution of the relative motion probability density plotted in the lateral plane of NPLs (lower row) *n* = 5.5 ML. *L*_*x*_ = 20 nm and *L*_*y*_ = 30 nm.

Fig. 2 schematically shows the qualitative behavior of the total interaction and electric field potential and probability density of the exciton relative motion as a function of the field value. It is clear from general quantum-mechanical considerations that in the asymmetric potential well from eqn (12) with an increase in the field, a certain moment comes when the existence of bound exciton states becomes impossible.^64^ This corresponds to the physical situation when the external field becomes so strong that it destroys the exciton as a bound state (Fig. 2c). An external field-induced dissociation of the exciton already occurred (Fig. 2c). This situation is observed for the fields larger than critical *F* > *F*^ex^_cr_ (see Table 2). This corresponds to the physical situation when the probability of particle localization inside the Coulomb well deformed by the external field is smaller than the corresponding probability of being outside the well. This criterion is used to calculate such a threshold value of the electric field *F*^ex^_cr_. The probability itself is calculated using the relative motion wave function, obtained by numerical solution of eqn (12). A more detailed change in the shape of the electron–hole interaction potential and the wave function of the exciton as a result of the numerical solution of eqn (12) is shown in Fig. 3.

**Table 2:** The critical values of the external field above which the bound exciton state in the nanoplatelet ceases to exist, and the corresponding field-dependence coefficient *a* (see (15)) for the Stark shift at *ε*_b_ = 2

*n*	*F* ^ex^_cr_ *ε*_b_ = 2 (kV cm^−1^)	*F* ^ex^_cr_ (*ε*_b_ = 5) (kV cm^−1^)	*F* ^ex^_cr_ (*ε*_b_ = 10) (kV cm^−1^)	*a ε*_b_ = 2 (meV cm^2^ V^−2^)
3.5	285	170	125	−78.9
4.5	245	155	110	−153.9
5.5	220	135	95	−260
7.5	185	125	90	−448

As we can see from Fig. 3, the external field introduces asymmetry into the interaction potential (8) and (9). The asymmetry increases with the external field, and accordingly, the electron and hole relative motion wave function is stretched in the direction of the field, and under strong fields, the exciton as a bound state ceases to exist. Such critical values are weakly dependent on lateral sizes for typical NPL lateral sizes; therefore, they depend on the monolayer number (see Table 2).

**Fig. 4:**
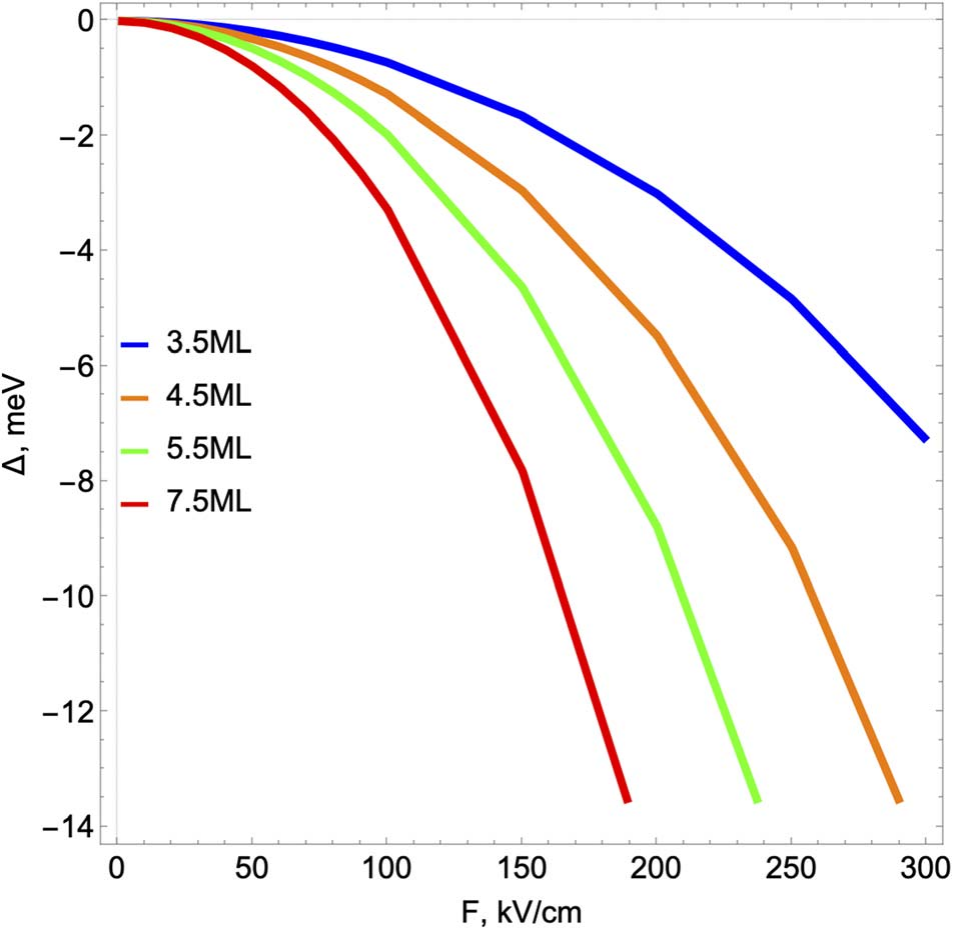
Stark shift dependence on the external electric field.

As we can see, with an increase in *ε*_b_, the dielectric confinement weakens as a result of which the critical value *F*^ex^_cr_ decreases. Let us now consider the behavior of the 2D exciton ground energy level in the external field. Fig. 4 graphically shows the Stark shift of the ground exciton level under the influence of an external field. A similar dependence of the Stark correction value on the external field is characteristic of exciton states in size-quantized structures.^47,48,56,65^ The binding energies of excitons in similar systems are practically of the same order of magnitude and amount to hundreds of meV.^56,65–68^ Accordingly, the calculated curve obtained by us for the same range of external field values repeats the general behavior of the Stark shift curves for a 2D exciton in the aforementioned studies. For weak fields, such behavior corresponds to the quadratic dependence of the Stark shift known from quantum mechanics Δ*E*(*F*) on the magnitude of the field strength:^44,56,65,67–69^

15 Δ*E*(*F*) = (*E*^b^_ex_(*F*))_1_ − (*E*^b^_ex_(*F* = 0))_1_=*aF*^2^

Moreover, both the magnitude of the shift itself and the growth rate of the curve increase with the increase in the number of atomic monolayers of NPLs in the direction of quantization. This is explained by the weakening of the binding energy of the 2D exciton with an increase in the thickness of the NPL and the corresponding increase in the influence of the external field on the exciton state. It should be noted that with an increase in the number of monolayers, the Stark shift values are decreasing, approaching corresponding values of this shift in the case of spherical quantum dots.^47,48^ This is due to the increase in the binding energy of the exciton in NPLs with a decrease in the number of monolayers.

## 3. Exciton lifetime in CdSe NPLs in the presence of an in-plane electric field

Let us turn to the consideration of the lifetime of exciton states in the presence of an external electric field. Under the action of the field, as is known, several processes occur that ultimately lead to the destruction of the 2D exciton. Each of these processes is characterized by its lifetime. An exciton, even without external influence, is a quasiparticle with a finite lifetime. At low exciton densities and temperatures not exceeding room temperature, the primary mechanism of exciton recombination is radiative recombination, which is described by *τ*_rad_ and can be calculated using the following expression:^70,71^16
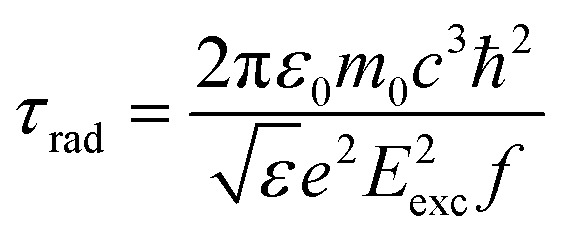
Here17
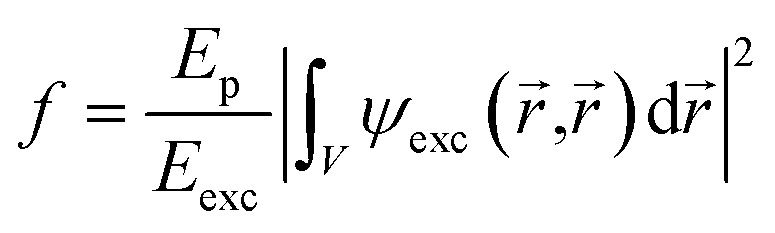


**Fig. 5:**
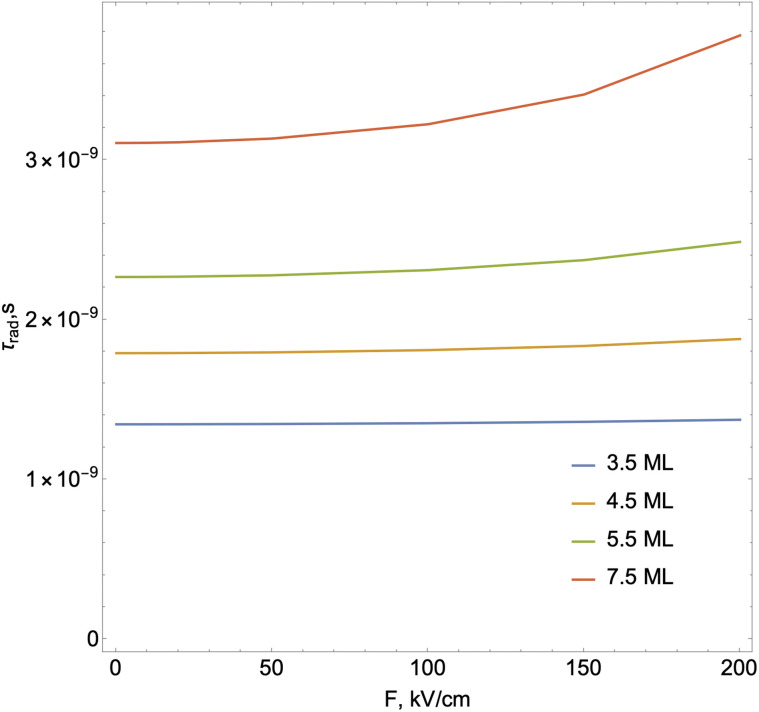
The exciton radiative lifetime dependence on the electric field for different numbers of monolayers.

In exp (17)*E*_p_ – is the Kane energy of an interband transition for CdSe *E*_p_ = 16.5 eV;^2^*E*_exc_ is the energy that corresponds to the interband transition from the valence band to the conduction band taking into account excitonic effects. In Fig. 5, the exciton radiative lifetime dependence on the electric field for different numbers of monolayers is shown.

**Fig. 6:**
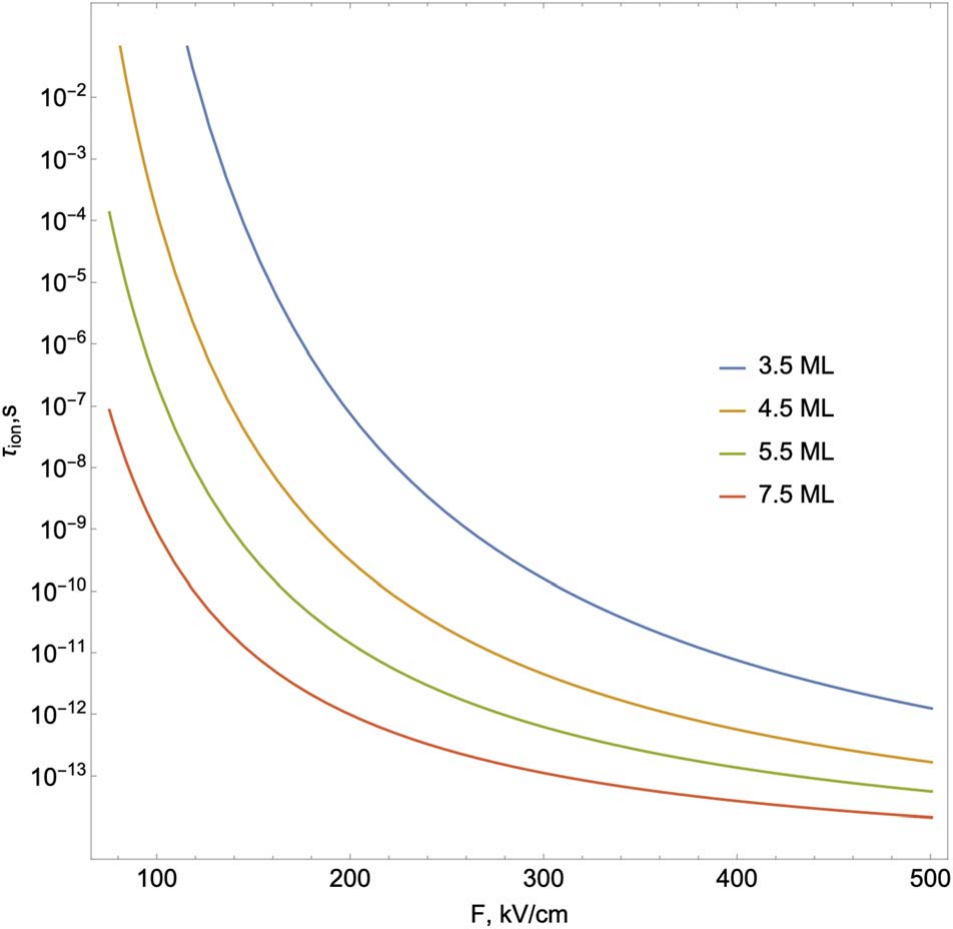
The ionization time dependence on the external electric field.

As we see in comparatively weak fields, the effect of the external field on the radiative lifetime of the exciton is not significant. However, with increasing field strength, the lifetime of the exciton still slowly increases. This is mainly due to the stretch of the electron and hole relative motion wave function along the field direction and the corresponding decrease in the value of *f*, which leads to an increase in the lifetime *τ* ∼ 1/*f*. At the same time, an increase in the magnitude of the transition energy is characterized by the Stark shift; however, this energy addition is not significant Δ*E*(*F*)≪*E*_exc_ and a dominating factor in the lifetime dependence is the reduction of the overlap integral of the wave function. A similar behavior for the exciton radiative lifetime is also observed in the case of spherical CdSe quantum dots in an external electric field.^47^ However, in our case, we observe a slow increase in the lifetime (decrease in the decay rate) due to much stronger coupling between electrons and holes in NPLs compared to the quantum dot case.^47^ On the other hand, with an increase in the number of monolayers, an increase in the lifetime of the exciton is observed. Analogous dependence of exciton radiative lifetime on the monolayer number is measured experimentally in a study.^72^ In this situation, a decisive role will be played by a significant decrease (hundreds of meV) in the energy of interband transitions due to a decrease in the confinement effect in the axial direction. At the same time, under the influence of the field, the overlap integral will decrease again, which in turn will increase the lifetime. As already noted, an exciton can be dissociated by the action of an external field. An increase in the value of the electric field will lead to an increase in the probability of tunneling and, consequently, the ionization time will decrease. The ionization time can be estimated with a quasi-classical approach using the following known formula:^73^18
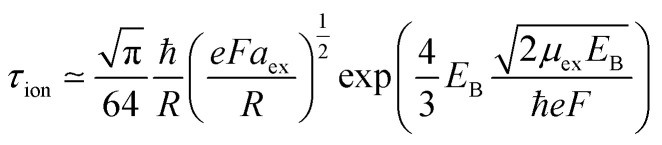
where *E*_B_ is the 2D exciton binding energy in the absence of an electric field defined as 
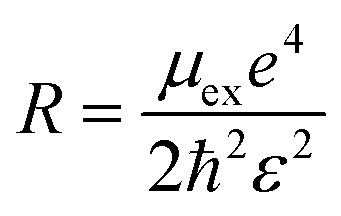
. Fig. 6 shows the exciton ionization time dependence on the external electric field.

**Fig. 7:**
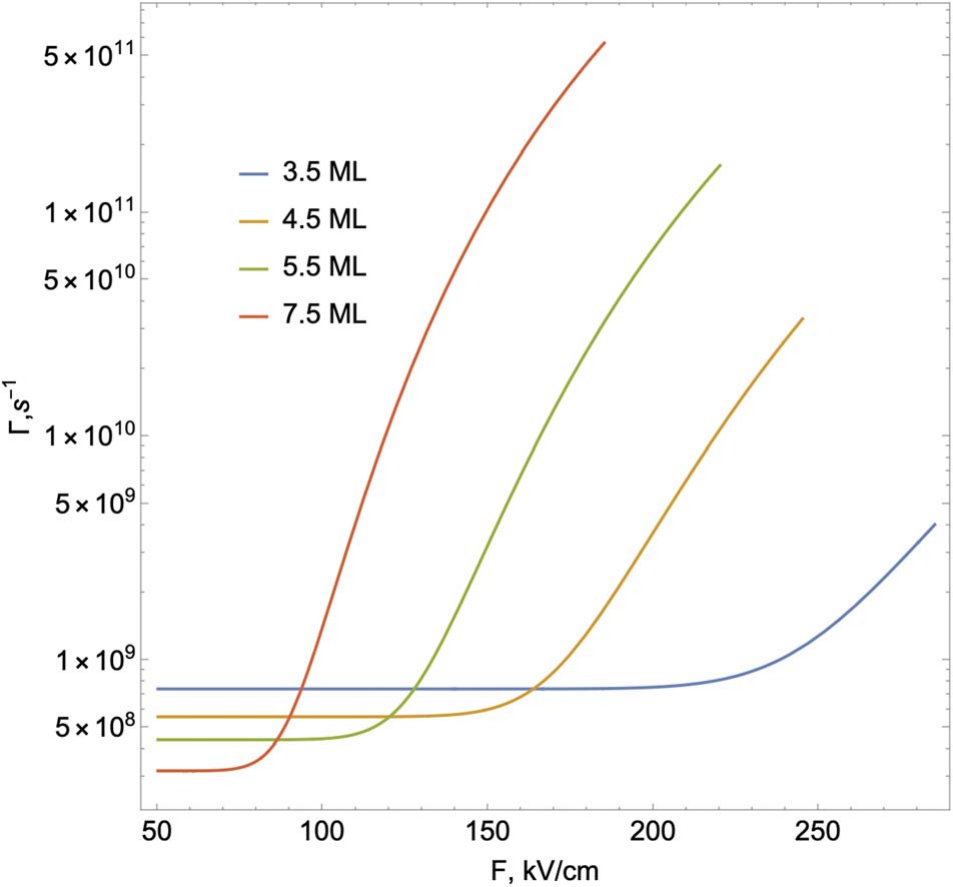
Theoretical total exciton decay rate in CdSe NPLs in the presence of an external field.

As shown in Fig. 6, ionization lifetime monotonically decreases (the probability of tunneling increases) with an increase the external field, as expected. The increased probability of tunneling can be attributed to the steeper gradient of the potential in the presence of an external field, which enhances the likelihood of quantum particles overcoming the potential barrier. Simultaneously, the ionization process initiates at lower electric field strengths with increasing NPL thickness. This is because with increasing thickness, the binding energy of the 2D exciton will decrease. Accordingly, the ionization process under the action of the field begins to manifest itself for smaller external field values. Considering both ionization and recombination lifetime results, one can calculate the total exciton decay rate (shown in Fig. 7):19
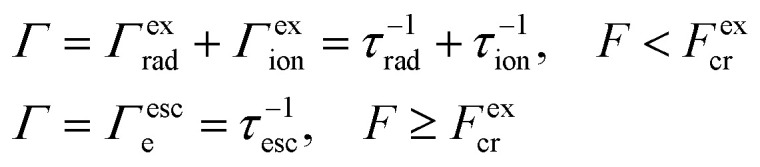


As can be seen from Fig. 7, for each NPL thickness, there is a critical value of the electric field up to which the exciton decay rate remains practically constant and after which it increases quite rapidly. Moreover, for small values of the fields, the exciton decay rate is determined by the recombination lifetime, and with the external field value increasing, the role of the ionization becomes more vivid, and the total decay rate is mostly determined by the ionization lifetime. This process is completed when the field reaches the corresponding critical value *F* = *F*^ex^_cr_. The analogous behavior for a similar range of electric field values has been experimentally demonstrated.^56^ As for the dependence of the decay rate on the number of monolayers, with the monolayer number increasing, the recombination lifetime increases (see Fig. 5), and correspondingly, the starting value of the decay rate becomes smaller. Also, with the increase in the number of monolayers, the slope of the exciton decay rate curve gets steeper. This is due to the difference in exciton binding energy depending on the number of monolayers.^56,73^

## 4. Free carrier states in the strong electric field

Let us now turn to free carrier states in NPLs in a strong electric field. After the exciton decays under the action of an electric field, the Coulomb interaction between the electron and hole can be neglected, and their state will be determined by the magnitude of the external field and the parameters of the NPL quantum well in the lateral direction. The depth of the well is determined by the relationship between the characteristics of the NPL material and the environment. If the CdSe NPL is surrounded by a dielectric material, then the depth of such a potential well is of the order of 500 meV for the electron,^63^ and if the NPL is surrounded by the so-called “crown” (CdSe/CdS) then the depth of the potential well is significantly less and is of the order of 200 meV.^3,74,75^

In this section we consider the motion of already free carriers in a strong field. At the same time, since the field acts along the *y* – direction, we do not consider the motion of carriers along the *x* coordinate. The external field outside the well creates a triangular potential barrier through which there is always a finite probability of tunneling. This probability naturally increases with the strength of the field. The probability of tunneling for the electron will be greater than for the hole in 
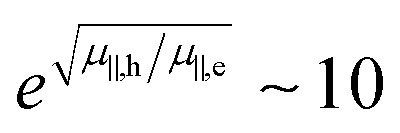
 times, because of the small value of the effective mass of the electron compared to that of the hole. Therefore, we will consider only the electronic states.

The behavior of the electron in the direction of the field in this case will be described by the following equation, taking into account the finiteness of the confining potential in this direction:20
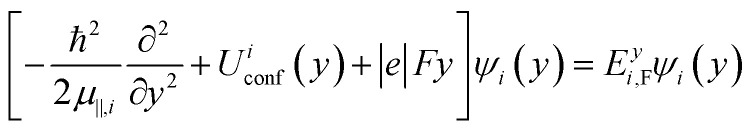
21
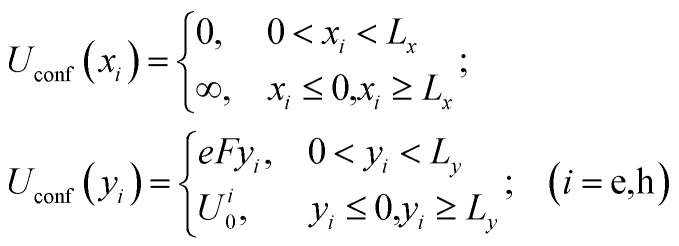
Here, *U*^*i*^_0_ – the depth of the potential well in the *y*-direction.

**Fig. 8:**
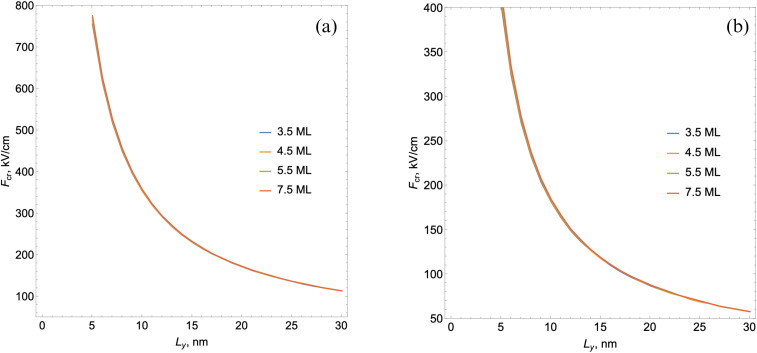
The behavior of the limiting value of the external field depending on *L*_*y*_ for different numbers of monolayers and quantum well depths *U*_0_ = 500 meV (a) and *U*_0_ = 250 meV (b).

With the subsequent increase in the external field's intensity, a moment comes when the discrete states in the well deformed by the external field no longer exist. Fig. 8 shows the behavior of the limiting value of the external field *F*^esc^_cr_ starting from which the absence of discrete electronic levels in the well is observed depending on the *L*_*y*_ and number of monolayers for quantum well depths *U*_0_ = 500 meV (a) and *U*_0_ = 250 meV (b).

**Fig. 9:**
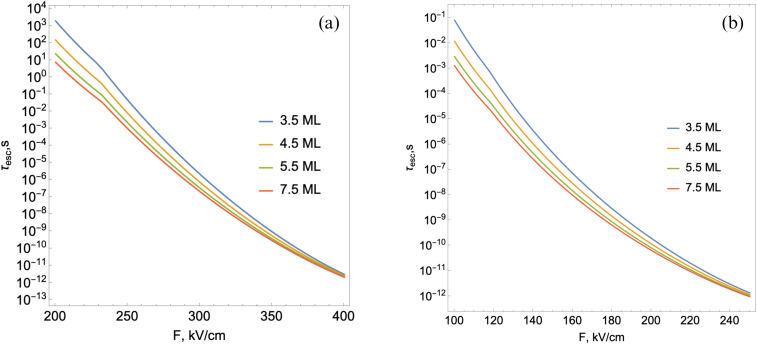
Electron escape time depending on the external field intensity for different values of monolayers and at a fixed value of *L*_*y*_ = 15 nm in the case of *U*_0_ = 500 meV (a) and *U*_0_ = 250 meV (b).

Fig. 8 demonstrates that the critical value of the electric field *F*^esc^_cr_ (from which the absence of a discrete electron state in the well is observed) decreases with an increase in the lateral size of the NPL. In contrast to the critical value of the field at which there is no exciton state *F*^ex^_cr_, it weakly depends on *L*_*y*_, and strongly depends on the number of monolayers. For field intensities smaller than the limiting value, as has already been noted, the existence of discrete levels with a certain tunneling probability is possible. We estimate the probability of electrons through the triangular barrier using the well-known formula for tunneling processes.^76^22
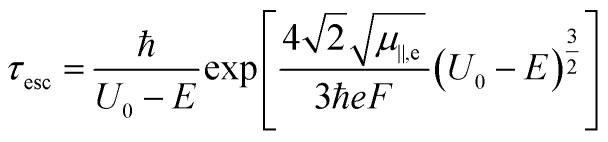
where *E* is the electron energy. As shown in Fig. 9, the electron lifetime associated with tunneling through the triangular barrier, depending on the electric field value, is demonstrated.

As can be seen from the graphs shown in Fig. 9, with an increase in the field value at a fixed value of *n*, the tunneling time decreases. This is due to both a decrease in the height and a narrowing of the width of the triangular barrier with an increase in the external field. With an increase in the number of monolayers, the effective mass of an electron in the lateral plane decreases, as a result of which the probability of tunneling increases. The possible mechanisms that affect lifetime in this strong electric field regime are the possible tunneling of charge carriers from the NPL region into the surrounding material as discussed above, and the recombination of free electron–hole pairs. The second process has a very low probability even at relatively small electric fields, as the free particles are localized at diametrically opposite sides of the NPL. To calculate the free carrier recombination time, we used the same methods as for calculating the exciton recombination lifetime with the difference that we do not put the interaction term in eqn (12) and we have to take into account all possible transitions for calculating oscillator strength in eqn (16) as the selection rules do not hold due to the external field. This means that this process will not affect the total lifetime in the strong electric field regime.

## 5. Conclusions

In summary, we have studied the excitonic state in CdSe NPLs in the presence of an external electric field. We have shown that, in the presence of an in-plane uniform field, a Stark shift of the exciton levels and subsequent ionization and decay of the bound electron–hole pair occur. Although the external field does not directly affect the states of carriers along the direction of strong quantization of the NPL (*z*), the thickness of the sample in this direction has a correlating effect on the binding energy of the 2D exciton: with an increase in the number of monolayers, the binding energy of the 2D exciton in the NPL decreases. As a result, in all processes considered in the work, the effect of the external field at a fixed value decreases with a decrease in the number of monolayers.

The critical value dependence of external field *F*^ex^_cr_ and *F*^esc^_cr_ on the geometrical parameters of NPLs has been identified: the critical field after which no bound exciton state exists decreases with the number of monolayers, and *F*^esc^_cr_ at which the electron has no localized state depends on the lateral NPL size. Exciton recombination and ionization determine the exciton decay rate in a weak field *via* tunneling lifetimes. The dependence of the exciton rate is nearly constant at small fields and has exponential growth up to the critical field value. These results agree with the experimental data for similar systems. After the decay of the exciton into a free electron and hole pair, the optical response is determined mainly by the electron tunneling out from the NPL.

## Conflicts of interest

The authors declare no conflicts of interest.
